# Effects of gastric bypass surgery on brain connectivity responses to hypoglycemia

**DOI:** 10.1007/s12020-022-03253-y

**Published:** 2022-12-02

**Authors:** Giovanni Fanni, Christakis Kagios, Erika Roman, Magnus Sundbom, Johan Wikström, Sven Haller, Jan W. Eriksson

**Affiliations:** 1 Department of Medical Sciences, Clinical Diabetes and Metabolism, Uppsala University, Uppsala, Sweden; 2 Department of Pharmaceutical Biosciences, Uppsala University, Uppsala, Sweden; 3 Department of Anatomy, Physiology and Biochemistry, Swedish University of Agricultural Sciences, Uppsala, Sweden; 4 Department of Surgical Sciences, Surgery, Uppsala University, Uppsala, Sweden; 5 Department of Surgical Sciences, Neuroradiology, Uppsala University, Uppsala, Sweden; 6 CIMC—Centre d’Imagerie Médicale de Cornavin, Geneva, Switzerland

**Keywords:** RYGB, fMRI, Feeding behavior, Counterregulatory response

## Abstract

**Introduction:**

Roux-en-Y gastric bypass (RYGB) leads to beneficial effects on glucose homeostasis, and attenuated hormonal counterregulatory responses to hypoglycemia are likely to contribute. RYGB also induces alterations in neural activity of cortical and subcortical brain regions. We aimed to characterize RYGB-induced changes in resting-state connectivity of specific brain regions of interest for energy homeostasis and behavioral control during hypoglycemia.

**Method:**

Ten patients with BMI > 35 kg/m^2^ were investigated with brain PET/MR imaging during a hyperinsulinemic normo- and hypoglycemic clamp, before and 4 months after RYGB. Hormonal levels were assessed throughout the clamp. Resting-state (RS) fMRI scans were acquired in the glucose-lowering phase of the clamp, and they were analyzed with a seed-to-voxel approach.

**Results:**

RS connectivity during initiation of hypoglycemia was significantly altered after RYGB between nucleus accumbens, thalamus, caudate, hypothalamus and their crosstalk with cortical and subcortical regions. Connectivity between the nucleus accumbens and the frontal pole was increased after RYGB, and this was associated with a reduction of ACTH (*r* = −0.639, *p* = 0.047) and cortisol (*r* = −0.635, *p* = 0.048) responses. Instead, connectivity between the caudate and the frontal pole after RYGB was reduced and this was associated with less attenuation of glucagon response during the hypoglycemic clamp (*r* = −0.728, *p* = 0.017), smaller reduction in fasting glucose (*r* = −0.798, *p* = 0.007) and less excess weight loss (*r* = 0.753, *p* = 0.012). No other significant associations were found between post-RYGB changes in ROI-to-voxel regional connectivity hormonal responses and metabolic or anthropometric outcomes.

**Conclusion:**

RYGB alters brain connectivity during hypoglycemia of several neural pathways involved in reward, inhibitory control, and energy homeostasis. These changes are associated with altered hormonal responses to hypoglycemia and may be involved in the glucometabolic outcome of RYGB.

## Introduction

Roux-en-Y gastric bypass (RYGB) is a surgical procedure that induces durable weight loss and has a positive impact on glucose homeostasis, lowering the risk of type 2 diabetes or even leading to diabetes reversal [1, 2]. This is achieved via gut anatomical and physiological rearrangements including hormonal adaptations that lead to enhanced incretin secretion and improved beta-cell function [3]. However, intriguing evidence suggests a much-underexplored role of the central nervous system in mediating the favorable effects of bariatric surgery [4].

Energy metabolism and food intake are finely regulated processes that rely on the hypothalamic integration of both central and peripheral signals [5]. However, the mesolimbic dopamine pathway (also known as the brain reward system) has been recently more and more recognized for its role in regulating glucose homeostasis [6], likely also via crosstalk with peripheral hormonal signals, including glucagon-like peptide 1 (GLP-1), insulin, ghrelin, and leptin [7–9].

The brain reward system is in engaged in the consumption of palatable food [10, 11], as well as by drugs of abuse [12]. Therefore, it is likely that aberrant eating behavior leading to obesity is related to morpho-functional abnormalities in the reward system neurocircuitry [11, 13, 14].

Neuroimaging techniques are versatile, non-invasive informative procedures and have highlighted changes in brain functional connectivity and glucose metabolism upon bariatric intervention. Several studies in the latest years have highlighted that after RYGB, resting-state connectivity in the fasting state is reshaped, especially as to brain regions part of the default-mode network (cingulate cortex, precuneus, basal ganglia), the prefrontal cortex, and the cerebellum [15–19]. Also, brain glucose uptake as assessed by ^18^F-FDG-PET in energy balance regions is altered after RYGB [20].

Our group was the first to perform a combined PET/MRI assessment under a hyperinsulinemic clamp with both a euglycemic and a hypoglycemic phase in patients before and after undergoing RYGB [21]. During the glucose-lowering phase, we found increased resting-state connectivity after surgery in an independent component including bilateral thalamus and hypothalamus, the brain’s central control unit of energy metabolism. Also, in a post-hoc analysis, we found that lateral hypothalamus connectivity was decreased towards the hippocampus and increased to the cerebellar vermis after RYGB [21]. This might reflect an RYGB-induced adaptation in neurocircuitries leading to a dampened response to hypoglycemia.

We hypothesize that brain connectivity after RYGB is reshaped in order to dampen the homeostatic response to energy deprivation. Therefore, in this study, we focused on RYGB-associated changes in regional brain connectivity during the glucose-lowering phase of the hyperinsulinemic clamp. We targeted brain regions that are of relevance for energy homeostasis and eating behavior according to previous work, including the thalamus, the ventral tegmental area, the basal nuclei, the amygdala, and the hypothalamus. The findings support a role of the brain in the favorable metabolic adaptations brought about by bariatric surgery.

## Methods

### Study design and subjects

Ten non-diabetic individuals with obesity (aged 25–49, BMI 35.2–45.4 kg/m^2^) were recruited at the obesity outpatient facility at Uppsala University Hospital during a routine visit before the scheduled Roux-en-Y gastric bypass (RYGB) operation. Further details of this cohort have been described and published before [21]. The subjects were investigated twice: approximately one month before RYGB and 4 months after RYGB. Also, according to local intervention guidelines, the patients underwent a 4-week low-calorie diet (800–1100 kcal/day) before surgical intervention. The study visits were performed at the PET/MR facility at Uppsala University Hospital and started at 8 am when the subjects were required to show up after an overnight fast. Medical history, anthropometric measures including total body fat assessed with bioimpedance, and blood samples were obtained.

### Investigations

At each visit, the subjects underwent a combined metabolic and neuroimaging assessment with simultaneous PET/MRI imaging and a two-step hyperinsulinemic clamp. This consisted of a first normoglycemic phase (5 mM) and a second hypoglycemic phase (2.7 mM). Also, cognitive assessments were performed during both the normoglycemic and the hypoglycemic phase using the Trail Making Test (TMT) and the Digital Symbol Substitution Test (DSST). Hypoglycemic symptoms were evaluated during hypoglycemia via the Edinburgh hypoglycemia symptoms score (EHSS). Blood samples were drawn from an antecubital vein after arterialization and were analyzed at the Department of Clinical Chemistry and the Clinical Diabetes Research Laboratory of Uppsala University Hospital. Neuroimaging was performed using an integrated PET/MR machine (SIGNA; GE Healthcare, Waukesha, WI), comprising a 3 T MRI scanner [21]. Details describing the steps of the whole combined procedure are described elsewhere [21]. During the clamp investigation, functional MRI (fMRI) scanning was run on three occasions: (1) during the normoglycemic phase; (2) during the glucose-lowering phase, and (3) during the hypoglycemic phase. Neuroimaging postprocessing details are described in supplemental materials of our previous publication [21].

### Neuroimaging exploratory analyses

For this post-hoc analysis, the CONN toolbox version 21.a [22] was used to identify changes after RYGB in regional connectivity of pre-determined seed regions of interest (ROI) to the whole brain, using a ROI-to-voxel approach. Anatomical demarcation of ROIs was performed with the embedded anatomic atlas of the CONN toolbox. Chosen seed regions were nucleus accumbens, caudate nucleus, putamen, medial and lateral hypothalamus, thalamus, amygdala, hippocampus, and globus pallidus. The software algorithm performed an automatic false discovery rate (FDR) correction of the results. All analyses were performed by averaging left and right hemispheres.

### Statistical analyses

Data are presented as mean and standard deviation (SD) or median and interquartile range (IQR) as appropriate and specifically indicated. Correlation analyses were performed with Pearson’s method after checking for normal distribution of the data using the Shapiro-Wilk test. Significance level was set at *p* = 0.05. Because of the exploratory nature of this investigation, correlation analyses were not corrected for FDR, and nominal p-values are given. Statistical analyses were performed using GraphPad Prism 9, and images were created using the CONN toolbox [22] and GraphPad Prism 9.

## Results

Using a ROI-to-voxel approach, we found several voxel clusters that displayed different connectivity after RYGB with brain regions that are relevant to energy homeostasis and feeding behavior (Table 1).

**Table 1:** ROI-to-voxel analysis with changes in connectivity after RYGB in the glucose-lowering phase of the hyperinsulinemic clamp

Seed region	Voxel cluster	Size	Anatomical region	*p*-value	Increase or decrease
Nucleus accumbens	+34 + 58 −18	221	Frontal pole right	0.006	Increase
	−08 −06 + 10	167	Thalamus right	0.012	Increase
	−30 + 44 −16	117	Frontal pole left	0.041	Increase
Caudate nucleus	−40 −38 −06	223	Middle temporal gyrus left (posterior division)	0.005	Increase
	+06 + 36 + 18	205	Cingulate gyrus (anterior division) Frontal pole left Paracingulate gyrus	0.005	Decrease
	+00 −46 + 42	177	Cingulate gyrus (posterior division) Precuneus	0.007	Decrease
Thalamus	+18 + 02 + 02	161	Pallidus right Caudate nucleus right Nucleus accumbens right	0.038	Increase
	+10 −82 −42	125	Cerebellum right (crus2, 7b, 8)	0.049	Decrease
Putamen	+44 −72 −30	841	Cerebellum right (crus1, crus2, vermis7)	0.000	Increase
	−52 −62 −34	401	Cerebellum left (crus1, crus2)	0.000	Increase
Lateral hypothalamus	−18 −38 −20	182	Cerebellum left (4 5) Parahippocampal gyrus left (posterior division) Hippocampus left	0.006	Increase
	+48 −36 + 58	176	Supramarginal gyrus right (posterior division) Postcentral gyrus right Supramarginal gyrus right (anterior division)	0.006	Decrease
	−06 −44 + 02	167	Cerebellum left (vermis 4 5, 4 5) Cingulate gyrus (posterior division) Precuneus	0.006	Increase
	+46 + 24 −24	151	Temporal pole right Frontal orbital cortex right Insular cortex right	0.008	Increase
	−26 + 24 −12	134	Frontal orbital cortex left Frontal pole left Putamen left	0.011	Decrease
Medial hypothalamus	+02 −58 −30	268	Cerebellum (vermis 8, 9, 4 5)	0.003	Increase
	+28 + 10 −30	167	N/A	0.027	Decrease
	+28 −34 + 34	146	Superior parietal lobule right	0.034	Decrease
Amygdala	NR				
Hippocampus	NR				
Globus pallidus	NR				

ROI anatomically defined according to CONN toolbox’s embedded anatomical atlas. Voxel clusters are defined according to X/Y/Z coordinates in the MNI space

*NR* no results

**Fig. 1:**
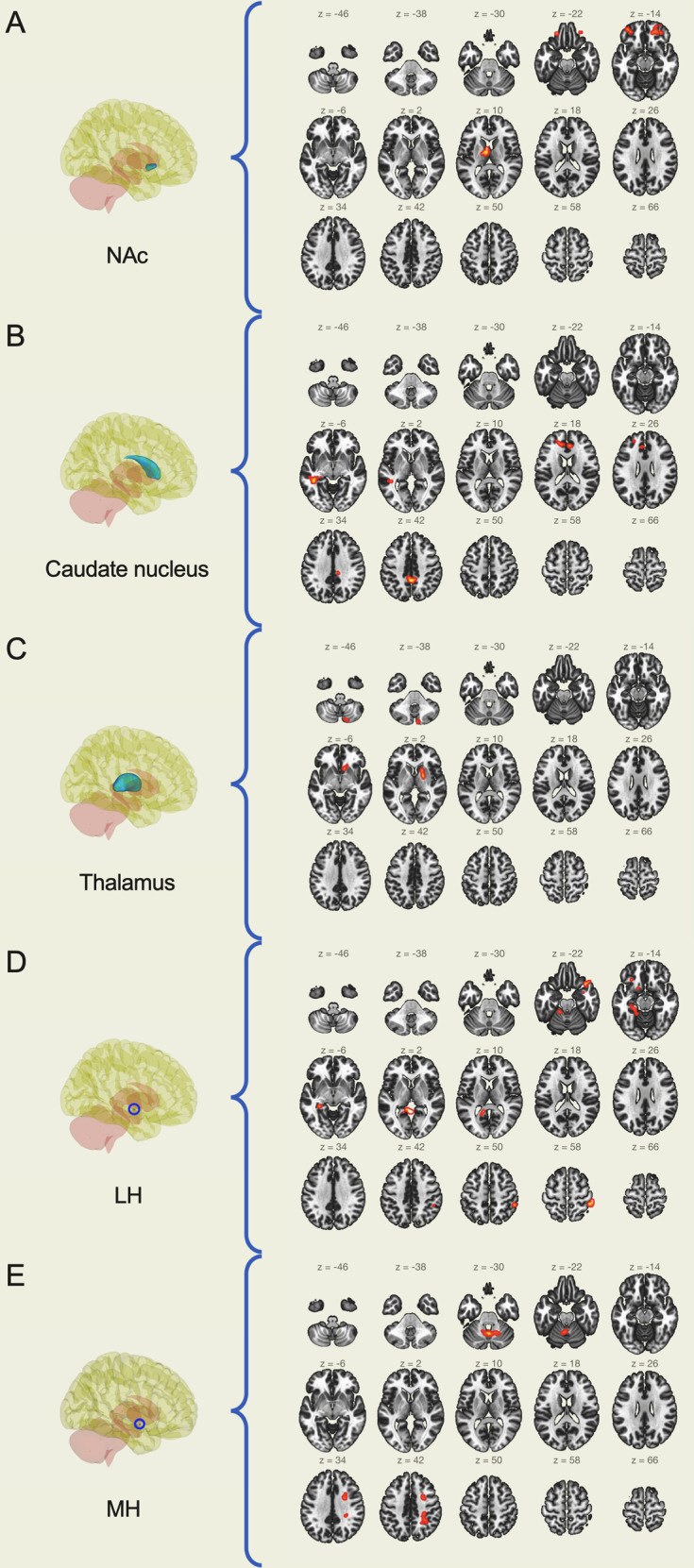
ROI-to-voxel analysis with changes in connectivity after RYGB in the glucose-lowering phase of the hyperinsulinemic clamp. The brain slice images (right part of the panel) show in red the voxel clusters that have significantly different connectivity with the seed region (depicted in blue on the left). **A** Nucleus accumbens, **B** caudate nucleus, **C** thalamus, **D** lateral hypothalamus, **E** medial hypothalamus. Created with the CONN toolbox

Connectivity of the nucleus accumbens with voxel clusters located in both left and right frontal poles and the right thalamus was increased (Fig. 1A). The caudate nucleus showed increased connectivity with the posterior division of the left middle temporal gyrus, and decreased connectivity with the cortex of the medial surface of brain hemispheres, including anterior and posterior cingulate and paracingulate gyrus, precuneus, and frontal pole (Fig. 1B). Thalamus connectivity was increased with a cluster located on the right side stretching over paleostriatum (globus pallidus), neostriatum (caudate nucleus) and ventral striatum (nucleus accumbens), while decreased with the right cerebellum (Fig. 1C). We found increased putamen connectivity with both right and left cerebellum. Lateral hypothalamus showed the greatest changes in connectivity after RYGB, with altered connectivity with several medial and lateral cortical areas, left hippocampus and parahippocampal gyrus, cerebellum, and the putamen (Fig. 1D). Connectivity of the medial hypothalamus was increased with the cerebellum and decreased with the right superior parietal lobule (Fig. 1E). On the contrary, regional connectivity of amygdala, hippocampus and globus pallidus were unaffected after RYGB (Table 1).

**Fig. 2:**
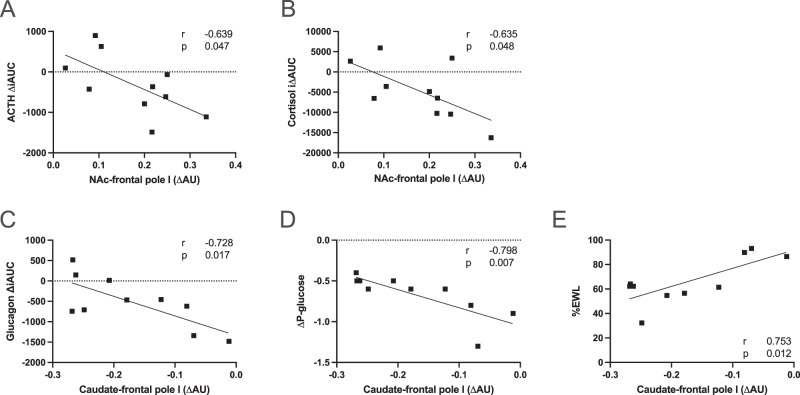
Pearson’s correlations between changes in regional connectivity and (**A**) change in the iAUC of ACTH, (**B**) change in the iAUC of cortisol, (**C**) change in the iAUC of glucagon, (**D**) fasting plasma glucose, (**E**) percentage excess body weight loss (%EWL)

Increased connectivity between the nucleus accumbens and the frontal pole was associated with reduced iAUC (incremental area under the curve) of both ACTH (*r* = −0.639, *p* = 0.047) and cortisol (*r* = −0.635, *p* = 0.048) during the hypoglycemic clamp (Fig. 2A, B).

However, connectivity between the caudate and the frontal pole after RYGB was reduced and this correlated with less reduction of the glucagon response during the hypoglycemic clamp (*r* = −0.728, *p* = 0.017 for iAUC changes; Fig. 2C), smaller reduction of fasting plasma glucose (*r* = −0.798, *p* = 0.007; Fig. 2D), as well as with lower percentage excess weight loss (%EWL) (*r* = 0.753, *p* = 0.012; Fig. 2E).

None of the other changes in regional connectivity was associated with %EWL, changes in BMI, fasting plasma glucose, waist-to-hip ratio, clamp M-value, body fat percentage, or the ∆iAUC of any other counterregulatory hormone (data not shown).

We have previously shown that cognitive function as assessed by psychometric tests correlated with cerebral blood flow during hypoglycemia, and that both of them were increased after RYGB [21]. However, these changes were not associated with any surgery-induced change in regional connectivity after RYGB (data not shown).

## Discussion

We found several resting-state neural pathways whose activity was altered after RYGB during the glucose-lowering phase of the hyperinsulinemic clamp, involving both basal nuclei and cortical regions. Some of these changes were also associated with surgery-induced changes in brain glucose metabolism and peripheral counterregulatory responses.

### Striatum and frontal cortex

In the mesolimbic dopamine pathway, the release of dopamine by neurons projecting from the ventral tegmental area onto dopamine receptors expressed by the GABAergic spiny neurons of the nucleus accumbens promotes the desire for rewarding stimuli, a process known as incentive salience [23]. Both short-projecting GABAergic neurons within the ventral tegmental area [24] and long-projecting GABAergic neurons to the shell of the nucleus accumbens [25] participate in the fine-tuning of this network. We found increased connectivity between the nucleus accumbens and the frontal pole, a region whose neurological function is still poorly understood, but seems to be involved in the so-called cognitive branching [26], that is the ability to attribute priority to different tasks scheduled to achieve an objective. Since efferent nucleus accumbens projections are GABAergic, thus inhibitory [27], we speculate that the increased connectivity between these regions during the glucose-lowering phase reflects a reduction in the cognitive response to energy deprivation. In addition, patients with the greatest increase in this connectivity also showed the most attenuated HPA-axis activation following RYGB. Altogether, these findings suggest that RYGB leads to intertwined readaptations of glucose homeostasis at several levels, affecting hormonal and cognitive responses, brain connectivity and glucose metabolism.

The connectivity between the nucleus accumbens and the thalamus, as parts of the reward system, is responsible for the translation of motivation inputs into motor responses [28]. We also found increased connectivity between these two regions after RYGB, suggesting an altered motivational incentive to food-seeking during hypoglycemia.

Connectivity between the caudate nucleus and the frontal pole, which operate together in behavioral inhibitory control [29] and goal-directed behavior [30], might also be relevant in behavioral adaptation to hypoglycemia after RYGB. Remarkably, patients with the greatest attenuations in this connectivity pathway had the least reduction in glucagon responses during the hypoglycemic clamp, less loss of excess weight and less improvement in fasting plasma glucose, suggesting a central compensatory mechanism to the great RYGB-induced metabolic adaptations.

### Default-mode network

A reduction in the posterior cingulate cortex and the precuneus functional connectivity is known to occur at fasting after gastric bypass [15–18]. Both the posterior cingulate cortex and the precuneus are key neural hubs of the default-mode network and play a role in internally directed attention and emotional salience [31, 32]. Deactivation of this region after RYGB is suggested to indicate reduced food-related salience attribution and reward-driven eating behavior [18]. Decreased connectivity of these regions with the caudate nucleus in the hypoglycemic phase suggests reduced salience attribution to the sense of hunger during energy deprivation after RYGB. Moreover, this system could also be directly influenced by homeostatic outputs from the lateral hypothalamus since we found increased connectivity of the latter with the posterior cingulate gyrus and the precuneus.

### Cerebellum connectivity

We found decreased connectivity of several brain regions, including caudate nucleus, putamen, medial hypothalamus, and thalamus, with the cerebellum, whilst connectivity of both medial and lateral hypothalamus with the cerebellum was increased. The cerebellum is implicated in excessive eating and satiation signals [33, 34]. Simultaneous transcranial stimulation of the prefrontal cortex and the cerebellum induced remarkable alterations in appetite and desire to eat in human obese individuals [35]. Our results support the role of RYGB in remodeling neurocircuitry between several subcortical regions and the cerebellum during hypoglycemia, in accordance with the emerging evidence that attributes the cerebellum a relevant role in feeding behavior and satiation [34].

### Thalamus connectivity

We found altered connectivity between the thalamus and the nucleus accumbens after RYGB. Paraventricular thalamic projections to the nucleus accumbens are important in motivated behaviors [28] and promote both feeding [36] and sucrose-seeking behaviors during hypoglycemia [37], as well as and high-fat diet preference [38] in murine models. Thus, RYGB might affect feeding behavior through modulating motivational aspects of feeding behavior, especially in conditions of energy deprivation, with improved metabolic aftermaths.

We also found increased connectivity of the thalamus with the caudate nucleus and the globus pallidus, a pathway implied in cognitive control on behavior [39].

### Hypothalamus connectivity

We found increased connectivity between the lateral hypothalamus and the hippocampus. A study carried out on patients with narcolepsy identified decreased connectivity between the lateral hypothalamus and the hippocampus, and speculated that this was a proxy for decreased orexin/hypocretin innervation, a neurotransmitter that is of great importance also in appetite regulation [40]. This circuitry featuring melanin-concentrating hormone neurotransmission is not only extremely relevant in orexigenesis but is also involved in impulsive behavior [41].

Recently, it has been shown that connectivity between the lateral hypothalamus and several cerebellar regions was associated with the weight loss outcome in obese subjects undergoing a 3-month diet [42]. Accordingly, we found increased connectivity in this pathway after RYGB, suggest its favorable role in weight control.

### Limitations

This study has some limitations. Firstly, it is an exploratory post-hoc investigation, so no formal power calculations were made and some of the analyses might therefore be underpowered. Secondly, this is an observational study, and no conclusions of causality can be made based on the presented results. Thirdly, the lack of a control group does not allow to distinguish between the effects of RYGB and the weight loss per se. However, several studies have highlighted the structural and functional changes following bariatric surgery in the human brain being independent of weight loss [43]. Finally, the sample size is small and true changes in other neural pathways may have been undetected due to lack of power as well as due to the ROI selection. Therefore, these findings should be used to generate hypotheses, and they need confirmation in additional studies.

## Conclusion

Altogether, these findings highlight profound changes following RYGB in brain cortical and subcortical connectivity during experimental glucose deprivation. The affected neural pathways are largely involved in reward, inhibitory control or energy homeostasis. Among them, increased connectivity between nucleus accumbens and the frontal pole was associated with attenuated counterregulatory hormonal responses to hypoglycemia. On the other hand, a reduction in caudate nucleus to frontal pole connectivity was associated with a lower magnitude of favorable effects on glucose metabolism and adiposity after RYGB. These findings on the caudate are paradoxical, and might suggest that they occur as an adaptive or compensatory phenomenon, rather than as a primary mediator of metabolic improvement.

The findings may support that gastric bypass surgery partly exerts its favorable effects on energy and glucose homeostasis via the central nervous system, where changes in functional connectivity can contribute to modulation of the complex interplay between behavioral responses and neuroendocrine adaptations.

Conversely, the highlighted neural pathways may also be of relevance also for the development of obesity, insulin resistance and type 2 diabetes, and further research is warranted, including individuals in different stages of overweight and dysglycemia.

## Data Availability

The data and study protocol are available upon request to the corresponding author.
